# Acute Bilateral Posterior Meniscal Root Tears in the Setting of a Noncontact Anterior Cruciate Ligament Rupture

**DOI:** 10.1155/2024/2021725

**Published:** 2024-09-12

**Authors:** Adam V. Daniel, Shayne R. Kelly, Patrick A. Smith

**Affiliations:** ^1^ Columbia Orthopedic Group, 1 S. Keene Street., Columbia, Missouri, USA; ^2^ Missouri Orthopedic Institute, 1100 Virginia Avenue, Columbia, Missouri, USA

## Abstract

Combined medial and lateral posterior meniscal root tears in the setting of an acute anterior cruciate ligament (ACL) rupture are extremely rare. The following case report demonstrates a high school football player who sustained a noncontact knee injury while performing a spin move at practice. The patient is a 17-year-old high school football defensive end who was presented to the clinic 1 week following the injury complaining of persistent knee pain with associated swelling, limited range of motion (ROM), and complaint of instability. During physical examination, the patient was found to have anterior cruciate laxity. Magnetic resonance imaging (MRI) demonstrated a complete midsubstance tear of the ACL and increased signal within the posterior horn of the medial meniscus with no obvious signs of pathology localized to the lateral meniscus. ACL reconstruction (ACLR) was performed and intraoperatively, both medial and lateral root tears were found. A standard bone patellar-tendon bone (BTB) autograft ACLR was performed with combined medial and lateral root repair utilizing a transtibial pull-out method for both. The clinical importance is root tears with associated ACL tears can be hard to diagnose on preoperative MRI, especially laterally, so careful assessment of both meniscal roots at the time of arthroscopy is critical. Furthermore, careful creation of the needed root repair tunnels for transtibial repair is critical to avoid coalescence with the ACL tibial tunnel.

## 1. Introduction

Meniscal injuries commonly occur in patients who sustain anterior cruciate ligament (ACL) tears [1, 2], particularly tears localized to the posterior horn [3, 4]. It is documented that lateral meniscal posterior horn root tears occur 10 times more commonly in the setting of an acute ACL injury compared to medial meniscal posterior horn root tears which are most commonly seen as degenerative meniscal lesions in middle-aged individuals [5, 6]. There is a paucity of literature describing acute bilateral meniscal root tears that occur together in the setting of an ACL injury. Additionally, there is no technical note to our knowledge that describes the treatment of this sequelae of pathology. The following case report presents a young, athletic patient who was treated for medial and lateral root tears in association with an acute ACL injury utilizing bilateral transtibial meniscal root repairs with separate tibial tunnels at the time of bone patellar-tendon bone (BTB) autograft ACL reconstruction (ACLR) with complete tibial tunnel.

## 2. Case Presentation

The patient is a 17-year-old male football player (defensive end), with no significant past medical history and a body mass index (BMI) of 27.7 kg/m^2^ who sustained a noncontact pivoting injury to his left knee while performing a spin move in practice (mechanism of injury: tibia external rotation, body internal rotation, valgus stress, and with the knee slightly flexed). He experienced immediate pain and swelling after he felt and heard an audible “pop” to his left knee while performing a spin move. Following the injury, he experienced left knee instability and was unable to continue playing. He utilized ice and over-the-counter anti-inflammatory medications with moderate relief and required crutches for mobilization. He initially presented to the clinic a week following the injury, but due to inadequate preoperative range of motion (ROM), he was prescribed prehabilitation and was seen 2 weeks following his initial visit. Although he did not have any additional injuries, at his second clinic visit, he complained of posteromedial knee pain and swelling.

### 2.1. Clinical Findings

On clinical examination, the left knee demonstrated a moderate 2+ effusion. ROM was from 0° short of full extension to 85° of knee flexion with complaints of pain and tightness. He had a 2+ positive Lachman as well as a 1+ positive anterior drawer with the foot in neutral and externally rotated positions. The posterior drawer was negative, and he was stable to varus and valgus stress testing at 30° of flexion. Pivot shift testing was unable to be performed due to pain and guarding. McMurray's testing was unable to be performed due to his limited flexion and guarding; however, he had focal tenderness over the posterior horn of the lateral meniscus. His patella was stable to lateral stress at 20° of knee flexion. Hamstring tightness and mild quadriceps atonia were also noted.

During the patient's second preoperative office visit—1 day prior to surgery—his knee was reevaluated. Joint swelling improved to a modest 1+ effusion. He still lacked 5° from full extension, but his flexion had improved to approximately 130°. It was also noted that he had tenderness to palpation around the posteromedial hamstring area in close approximation to the posterior horn of the medial meniscus, consistent with a newly formed palpable Baker's cyst. The remainder of the exam was similar to his first clinic visit.

### 2.2. Diagnostic Test Findings

**Figure 1:**
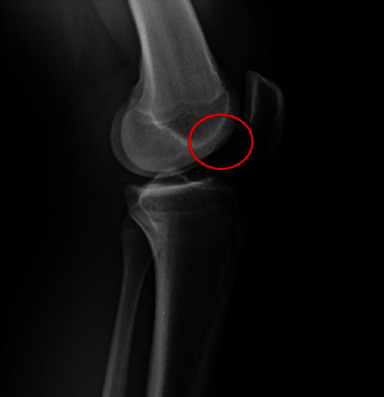
Lateral X-ray of a skeletally mature individual demonstrating a positive sulcus sign (red circle) suggestive of acute ACL injury.

KT-1000 arthrometer (Medmetric; San Diego, CA) testing demonstrated a 5-mm laxity at the 30-lb pull and a 7-mm of laxity at the manual maximum pull. Radiographs obtained at the office demonstrated closed growth plates as well as a positive sulcus with the lateral femoral condyle (Figure 1).

**Figure 2:**
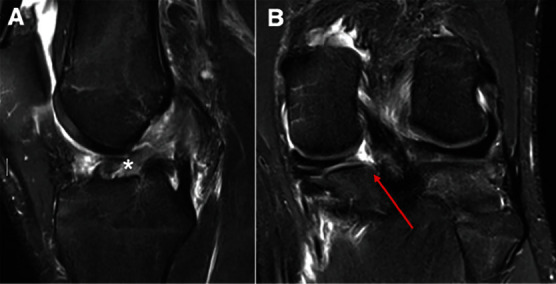
T2 MRIs of the patient's left knee demonstrating a complete midsubstance ACL tear on the (A) sagittal view (asterisk) as well as increased signal at the medial meniscal root attachment site (red arrow) on the (B) coronal view.

Magnetic resonance imaging (MRI) from an outside clinic demonstrated a complete midsubstance tear of the ACL (Figure 2(A)) as well as increased signal at the posterior horn of the medial meniscus with concern for root involvement (Figure 2(B)).

Despite the clinical tenderness over the posterior horn of the lateral meniscus, there did not appear to be any pathology noted within the posterior horn of the lateral meniscus on the MRI. The knee was otherwise ligamentously intact with the only other additional pathologic finding being a mild bone contusion involving the posterior aspect of the lateral tibial plateau.

### 2.3. Surgical Intervention

**Figure 3:**
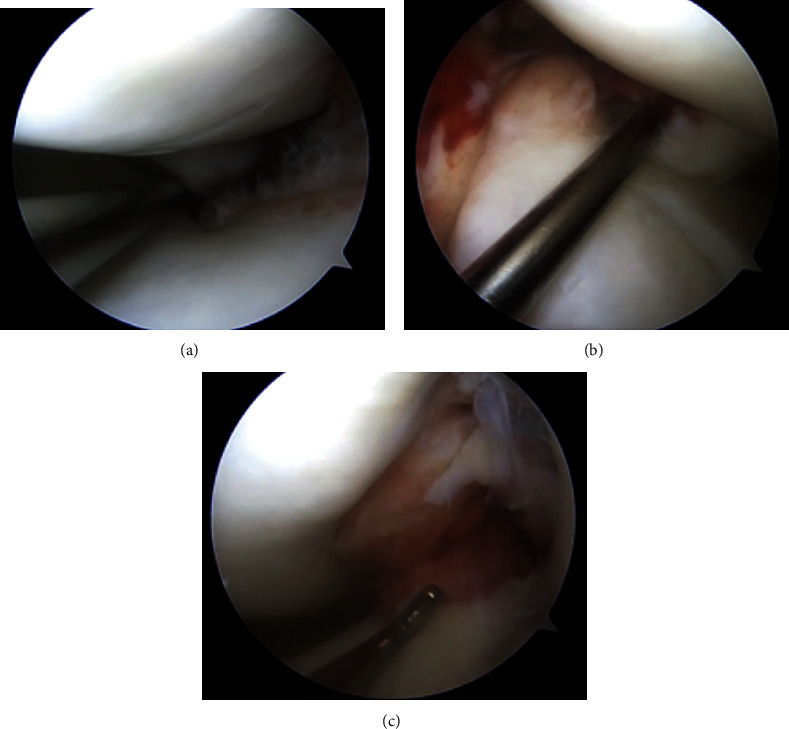
Arthroscopic view from the anterolateral portal of the (a) medial and (b) lateral meniscal root tears and (c) a midsubstance ACL tear.

**Figure 4:**
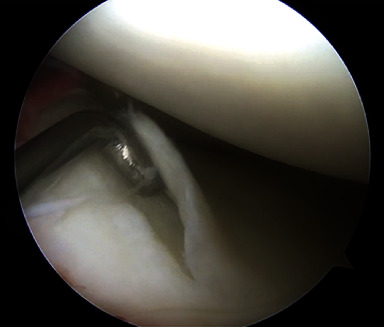
Unstable Grade 2 chondral lesion of the lateral tibial plateau.

Following a thorough diagnostic arthroscopy, the patient was found to have medial and lateral meniscal root tears as well as a midsubstance ACL tear (Figures 3(a), 3(b), and 3(c)). Additional pathology included an unstable Grade 2 chondral lesion at the posterior aspect of the lateral tibial plateau (Figure 4).

Prior to addressing the intra-articular pathology, an 11-mm-wide-by-5-mm-thick BTB graft was harvested and prepared for a complete tibial tunnel ACLR with suture tape augmentation.

Prior to addressing the lateral meniscal root tear, a shaver was used to debride the unstable lateral tibial plateau chondral lesion. An intraoperative plan was then developed relative to the order of tibial tunnel creation, in light of the fact that the patient needed transtibial lateral and medial root repair sockets and a full tibial tunnel, to avoid tunnel coalescence. First, the lateral meniscal root attachment site was debrided with a shaver to roughen up the bone for bleeding purposes. An ACL tibial aiming device was used to create the lateral root tibial tunnel using an all-in-one guide pin and retro reamer (FlipCutter; Arthrex, Naples, FL). After the guide pin was appropriately placed at the anatomic attachment of the lateral meniscus root, the FlipCutter was then expanded to 6 mm which was used to retro-cut back through the tunnel to create a 12-mm bone socket. Next, a shuttle stitch (TigerWire; Arthrex, Naples, FL) along with its shuttling sheath (TigerStick; Arthrex, Naples, FL) was introduced through this tunnel and retrieved through the anteromedial portal with the sheath discarded. Next, two #0 FiberLink; Arthrex, Naples, FL) were passed via an arthroscopic suture passer (Knee Scorpion; Arthrex, Naples, FL) in the lateral meniscal root with each suture converted to a cinch type configuration ensuring good purchase in the unstable root tissue [7]. The shuttle stitch was used to shuttle these two lateral meniscus sutures distally out the tibia to be fixed later on the tibia along with the suture tape (FiberTape; Arthrex, Naples, FL) used as the internal brace (InternalBrace; Arthrex, Naples, FL) for the ACLR construct.

Attention was then directed to the medial root. The medial root attachment site was prepared using a shaver in the same manner as the lateral root attachment site. Prior to creating the tunnel, trephination was performed over the superficial medial collateral ligament (sMCL) with an 18-gauge spinal needle to allow ligament lengthening to improve the visualization of the medial meniscus and to prevent iatrogenic injury to the cartilage. Three cinch configuration sutures were passed through the medial meniscus root ensuring good purchase with the all-arthroscopic suture passer [7, 8].

**Figure 5:**
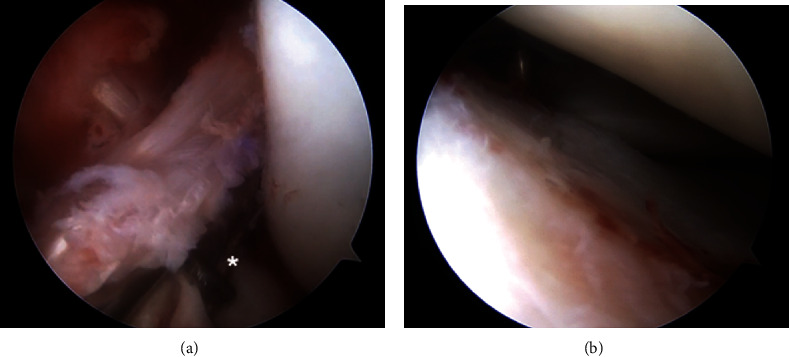
Arthroscopic view from the anterolateral portal of (a) the reconstructed ACL using a BTB autograft with suture tape augmentation (asterisk) and of (b) the reduced lateral meniscus following the transtibial repair. Of note, the sutures used to repair the lateral root and the suture tape used for the internal brace of the ACL graft were both fixed distally on the tibia using the same anchor (not pictured).

With care to avoid the lateral meniscal root repair tunnel, an 11-mm ACL tunnel drill (RetroDrill with 11-mm RetroCutter; Arthrex, Naples) was used to form a complete tibial tunnel for the ACL graft. The medial root tibial tunnel was made after the ACL tunnel to ensure no convergence. A shuttle suture in its sheath (TigerStick; Arthrex, Naples, FL) was passed and retrieved on the joint side to shuttle the three medial root sutures out of the tibia anteriorly. The ACL graft was fixed proximally using suspensory adjustable loop fixation (BTB TightRope II; Arthrex, Naples, FL). Final fixation of the internal brace and two lateral meniscus root repair sutures was done using a 4.75-mm anchor (BioComposite SwiveLock; Arthrex, Naples, FL) with the knee held in full hyperextension prior to final graft fixation (Figures 5(a) and 5(b)).

**Figure 6:**
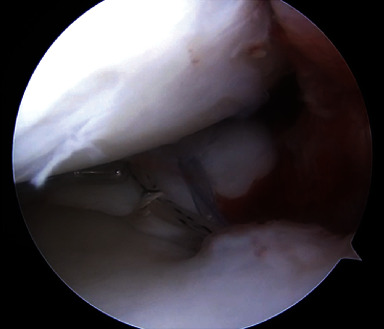
Arthroscopic view from the anterolateral portal demonstrating a reduced medial meniscus following transtibial root repair.

The BTB graft was then fixed distally using a 10-mm-diameter-by-20-mm-long interference screw (BioComposite Interference Screw; Arthrex, Naples, FL) again with the knee in full hyperextension. Finally, the three-cinch medial meniscal root repair sutures were fixed outside of their tunnel with the knee held at approximately 40° of flexion using a 4.75-mm anchor (Figure 6). The knee was then cycled approximately 15-20 times followed by retensioning of the ACL graft utilizing the femoral tightrope suspension fixation.

Knee stability was checked following the final graft retensioning. The patient had a negative Lachman, anterior drawer, and pivot shift. The ACL graft was arthroscopically inspected demonstrating a well-placed graft with the internal brace properly placed within the posterior portion of the graft and noted to be lax in flexion. Taking the knee through a full ROM confirmed the graft was in good position with no impingement from the notch even in full hyperextension. Both compartments were assessed and demonstrated anatomical reduction of both meniscal roots.

### 2.4. Follow-Up and Outcomes

The complete patient timeline is summarized in Table 1. Postoperatively, the patient was started on immediate ROM through use of continuous passive motion machine (KinexCONNECT; Kinex Medical Company, Waukesha, WI) in the recovery room which he then used at home for a minimum of 8 hours a day for the first 2 weeks after surgery. He was kept non-weight-bearing for 6 weeks [9].

Table 2 summarizes the physical exam findings for all the patient's postoperative office visits. All clinical parameters showed improvement throughout the postoperative period thus far. The patient has been neurovascularly intact in all subsequent visits. The patient has full extension and flexion and remains ligamentously stable with a negative Lachman and pivot shift. The patient does not have joint-line tenderness and McMurray's maneuvers are negative. Also, he has no palpable posteromedial Baker's cyst. Quadriceps atrophy has been evident throughout the follow-up period but has much improved since initial presentation and is now comparable to the contralateral leg. KT-1000 arthrometer testing at 1-year follow-up demonstrated no degree of increased anteroposterior laxity compared to contralateral knee.

Table 3 summarizes the patient's milestone progression throughout the postoperative period. For the first 3 months, the patient attended PT 3 times a week but following his 4-month visit was downgraded to 2 times a week.

**Figure 7:**
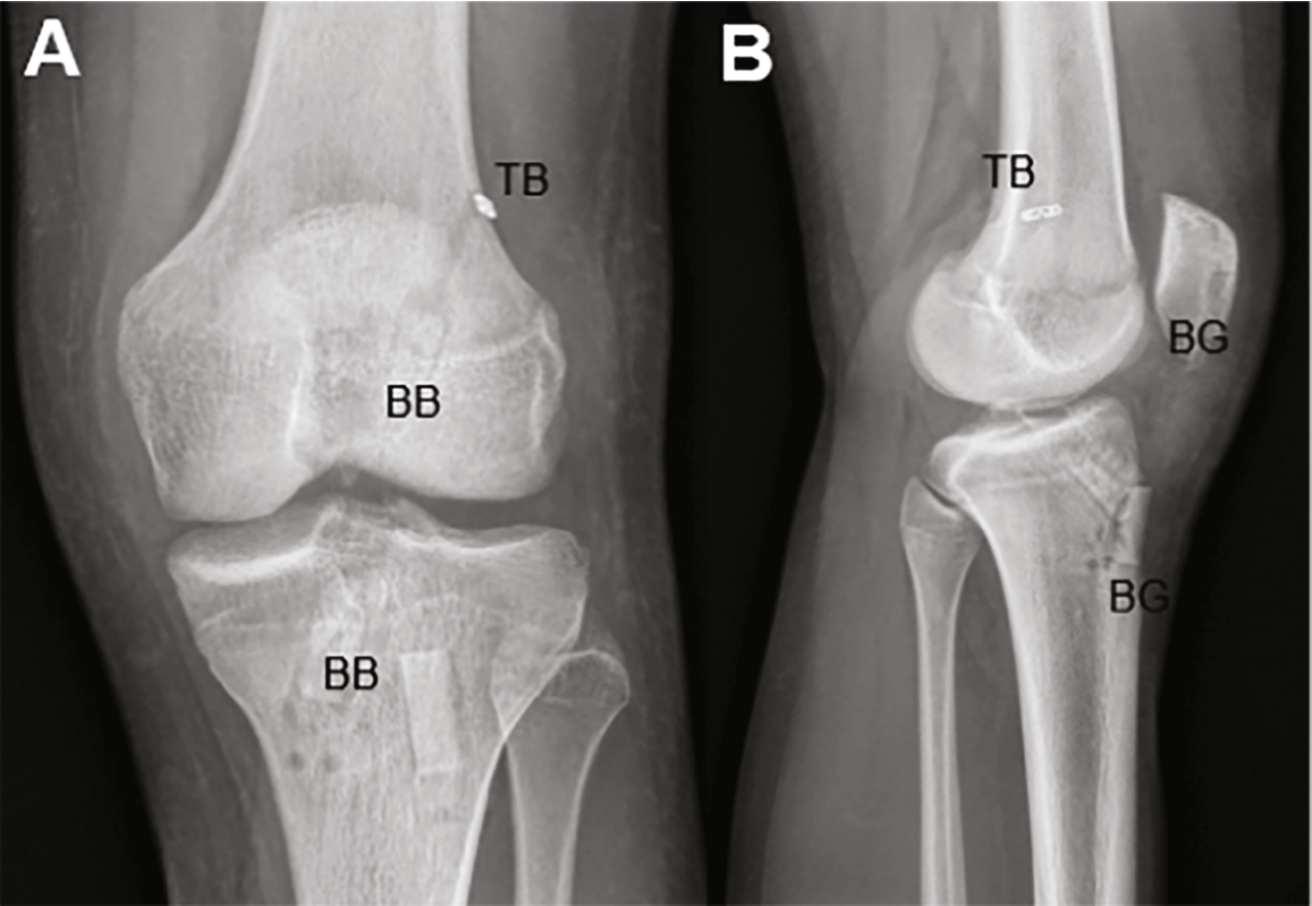
Left knee radiographs taken 2 weeks following BTB autograft ACL reconstruction with suture tape augmentation and bilateral transtibial root repairs. (A) AP radiograph of the patient's left knee demonstrating good bone block placement in the femur and tibia as well as good position of suspensory fixation button placement on the outer femoral cortex. (B) Lateral radiograph of the patient's left knee demonstrating well-placed bone grafts in the tibia and patella. TB = tight rope button; BB = bone block; BG = bone graft.

Radiographs were obtained during his 2-week postoperative visit which demonstrated good position of the ACL bone blocks and the suspensory fixation button on the lateral femoral cortex (Figures 7(A) and 7(B)). Bone grafting can also be appreciated in the patella and tibia from graft harvest sites.

## 3. Discussion

Posterior horn meniscal root tears may occur in the setting of an acute ACL tear, with tears of the lateral meniscal root occurring more often in the acute setting [3–6]. Although medial meniscal root tears may occur in the setting of an acute ACL injury, they are more often a result of chronic degenerative changes in an ACL-intact knee [5, 6]. There is a paucity of literature describing combined medial and lateral root tears occurring concomitantly in the setting of a noncontact, pivoting ACL injury.

Not only do the menisci function as a cartilage protectant and force distributor, they also secondarily stabilize the knee at certain degrees of knee flexion [10, 11]. Therefore, it is imperative to repair these lesions in the setting of an ACL tear not only to protect the articular cartilage and minimize the risk of later osteoarthritis but also to alleviate excess strain placed on the ACL graft itself [10, 11]. Additionally, it is preferred to repair meniscal root lesions in the setting of an ACLR because the intra-articular bleeding that typically accompanies ACLR may foster an ideal environment for healing of meniscal root repairs [12].

In this case, it was decided to repair both meniscal roots using the transtibial pull-out method as this is the standard of care for these lesions [6–8]. A major concern though in this scenario is the risk of tunnel convergence which may complicate the procedure [13]. Therefore, it is extremely important to visualize where the tunnels will be located intraoperatively and to plan the order of drilling with care to avoid inadvertently transecting fixation sutures, compromising the repairs. In this case, all three tunnels were drilled through the same medial tibial incision with the lateral root tunnel first so it could be avoided by the ACL tibial tunnel. Then, this allowed for safe drilling of the medial root tunnel as the last step to clearly avoid the other two tunnels.

This case report highlights the possibility that an acute bilateral posterior root injury may result in the setting of a noncontact, pivoting ACL injury. Although an important adjunctive diagnostic test—a MRI must complement the history and physical examination and not replace it. Particularly in this case, the physical examination elicited potential pathology associated with a posterior lateral meniscal root injury which was not well-visualized on the MRI [14]. Though the constellation of combined medial and lateral root tear pathology in this case is unique, the incidence may be globally underreported secondary to inadequate preoperative exam or lack of MRI findings which may have possibly led to a less-than-thorough diagnostic arthroscopy. The other clinical takeaway from this case report is formulating a good plan intraoperatively when confronted with unusual pathology before just moving straight to drilling the needed ACL tunnels without considering the risk of compromising root fixation.

This report is not without limitations. First, this case occurred relatively recently; therefore, it currently is not possible to assess the long-term outcomes following the combined medial and lateral root repairs and ACLR. Additionally, due to the novelty of the presenting pathology, an ideal technique for it has not been thoroughly described. Though we believe that the lesions were appropriately addressed, it may be possible that other operative techniques may yield similar results with less concern regarding tunnel convergence.

## 4. Conclusion

The clinical importance is that acute root tears with associated ACL tears can be hard to diagnose on clinically and on preoperative MRI, so careful assessment of both meniscal roots at the time of arthroscopy is critical. Therefore, surgeons should be ready to encounter and deal with posterior meniscus root pathology in every case undergoing ACLR by keeping all the necessary instruments and technical skill at disposal. Furthermore, careful creation of the needed root repair tunnels for transtibial repair is critical to avoid coalescence with the ACL tibial tunnel.

### 4.1. Clinical Message

Acute tears bilateral posterior meniscal roots in the setting of a noncontact ACL injury are extremely rare. A thorough physical examination may identify pathology that was not present on the MRI.

## Figures and Tables

**Table 1:** Patient timeline.

	**Patient timeline**
Initial injury	Patient reports hearing an audible “pop” in his left knee associated with immediate pain, swelling, and difficulty with ambulation following a noncontact pivoting maneuver while in football practice.
1 day following injury	Patient was seen by his primary care doctor who obtained an X-ray during the visit; he was scheduled to receive an MRI and was referred to our office.
3 days following injury	Patient received an MRI.
7 days following injury	Patient's initial clinic visit where he was scheduled for surgery. Prehabilitation was prescribed to improve range of motion prior to surgery.
20 days following injury	Patient reported to the clinic for a recheck prior to surgery to assess range of motion.
21 days following injury	Patient undergoes ACLR with bilateral transtibial root repairs.
	
Postoperative day 0	Patient was sent home with a CPM machine to facilitate early passive range of motion.
Postoperative day 3	Patient attended his first supervised PT session.
Postoperative day 15	Patient's first postoperative office visit.
Postoperative week 11	Patient's second postoperative office visit.
Postoperative month 4	Patient's third postoperative office visit.
Postoperative month 6	Patient's fourth postoperative office visit.
Postoperative month 12	Patient's final postoperative office visit.

**Table 2:** Postoperative effusion, range of motion, and quadriceps quality.

**Finding**	**2 weeks**	**11 weeks**	**4 months**	**6 months**	**9 months**	**1 year**
Effusion	1+	1+	Trace	Negative	Negative	Negative
Range of motion	0–80°	0–135°	0–135°	0–135°	0–135°	0–135°
Extensor lag^a^	2–3°	2°	None	None	None	None
Quadriceps tone	Good	Good	Great	Great	Great	Great

^a^Extensor lag was measured while patient performed straight leg raise.

**Table 3:** Postoperative milestones.

**Postoperative time point**	**Milestone**
Day 3	- Patient begins supervised physical therapy with focus on quadriceps strengthening to achieve full active knee extension.
Week 2	- Patient returns CPM machine. - Patient can now begin riding the stationary bike to help facilitate return of normal knee flexion.
Week 6	- Patient weaned off crutches. - Patient can now begin closed-chain exercises once good leg control is established.
Week 11	- Patient can now begin limited terminal knee extensions. - Blood flow restriction was implemented to facilitate straight leg raise without residual lag. - Patient can now begin to leg press but to avoid deep flexion.
Month 3	- Patient has achieved degrees of hyperextension during his physical therapy sessions.
Month 4	- Patient can now walk without a limp. - In addition to leg press and hamstring curls, the patient can now begin 45° mini-squats.
Month 6	- Blood flow restriction to help build muscle mass. - Continue open chain exercises. - Patient to start using the elliptical, may progress to jogging in 2–4 weeks.
Month 7	- Patient started using AlterG anti-gravity treadmill.
Month 9	- Patient cleared for agility training while wearing functional brace.
Month 12	- Patient to schedule appointments as needed.

## Data Availability

The authors have nothing to report.

## References

[1] Cain E. L., Fleisig G. S., Ponce B. A. (2017). Variables associated with chondral and meniscal injuries in anterior cruciate ligament surgery. *The Journal of Knee Surgery*.

[2] Michalitsis S., Vlychou M., Malizos K. N., Thriskos P., Hantes M. E. (2015). Meniscal and articular cartilage lesions in the anterior cruciate ligament-deficient knee: correlation between time from injury and knee scores. *Knee Surgery, Sports Traumatology, Arthroscopy*.

[3] Anderson M. J., Browning W. M., Urband C. E., Kluczynski M. A., Bisson L. J. (2016). A systematic summary of systematic reviews on the topic of the anterior cruciate ligament. *Orthopaedic Journal of Sports Medicine*.

[4] Tsujii A., Yonetani Y., Kinugasa K. (2019). Outcomes more than 2 years after meniscal repair for radial/flap tears of the posterior lateral meniscus combined with anterior cruciate ligament reconstruction. *The American Journal of Sports Medicine*.

[5] Matheny L. M., Ockuly A. C., Steadman J. R., LaPrade R. F. (2015). Posterior meniscus root tears: associated pathologies to assist as diagnostic tools. *Knee Surgery, Sports Traumatology, Arthroscopy*.

[6] Pache S., Aman Z. S., Kennedy M. (2018). Meniscal root tears: current concepts review. *Archives of Bone and Joint Surgery*.

[7] Smith P. A., Bley J. A. (2017). Simplified arthroscopic lateral meniscal root repair involving the use of 2 cinch-loop sutures. *Arthroscopy Techniques*.

[8] Woodmass J. M., Mohan R., Stuart M. J., Krych A. J. (2017). Medial meniscus posterior root repair using a transtibial technique. *Arthroscopy Techniques*.

[9] Calanna F., Duthon V., Menetrey J. (2022). Rehabilitation and return to sports after isolated meniscal repairs: a new evidence-based protocol. *Journal of Experimental Orthopaedics*.

[10] Tang X., Marshall B., Wang J. H. (2019). Lateral meniscal posterior root repair with anterior cruciate ligament reconstruction better restores knee stability. *The American Journal of Sports Medicine*.

[11] Uffmann W., ElAttrache N., Nelson T. (2021). Posterior lateral meniscal root tears increase strain on the reconstructed anterior cruciate ligament: a cadaveric study. *Arthroscopy, Sports Medicine, and Rehabilitation*.

[12] de Girolamo L., Galliera E., Volpi P. (2015). Why menisci show higher healing rate when repaired during ACL reconstruction? Growth factors release can be the explanation. *Knee Surgery, Sports Traumatology, Arthroscopy*.

[13] Campbell A., Narvaez M., Caldwell J. M., Banffy M. (2021). Stay ipsilateral: an analysis of tibial tunnel distance between cruciate ligament reconstruction and posterior meniscal root repair. *Arthroscopy, Sports Medicine, and Rehabilitation*.

[14] Krych A. J., Wu I. T., Desai V. S. (2018). High rate of missed lateral meniscus posterior root tears on preoperative magnetic resonance imaging. *Orthopaedic Journal of Sports Medicine*.

